# The amphiregulin- epidermal growth factor receptor axis as a therapeutic target in sepsis

**DOI:** 10.3389/fimmu.2025.1638244

**Published:** 2025-09-23

**Authors:** Rebecca Walsh, Timothy Arthur Chandos Snow, Alexandra Z. Martinez, David Brealey, Mervyn Singer, Abhishek Das, Nishkantha Arulkumaran, University College London Hospitals Critical Care Research Team

**Affiliations:** ^1^ Bloomsbury Institute of Intensive Care Medicine, University College London, London, United Kingdom; ^2^ NIHR UCLH Biomedical Research Centre, London, United Kingdom; ^3^ Division of Infection and Immunity, University College London, London, United Kingdom

**Keywords:** sepsis, intensive care unit, amphiregulin, epidermal growth factor receptor, monocyte, lymphocyte

## Abstract

The Epidermal Growth Factor Receptor (EGFR) and its ligand, amphiregulin (AREG) are critical for epithelial cell proliferation but their important role in inflammation and infection is increasingly described. We recently discovered that the EGFR ligand, amphiregulin, could identify a cohort of infants with sepsis, even when CRP was low, identifying it as a potentially adjunctive biomarker for early infection. Its role in adult sepsis however, has yet to be delineated. We conducted a prospective observational study of 42 critically ill septic adult patients on the Intensive Care Unit, comparing serum amphiregulin levels and the frequencies of EGFR-expressing myeloid and lymphoid cells (using spectral flow cytometry) in sepsis survivors and non-survivors, and 20 healthy volunteers. We demonstrate, for the first time, the strong association between serum amphiregulin and in-hospital mortality (AUROC = 0.87). Moreover, we demonstrate that a higher frequency of circulating CD4^+^ and CD8^+^ lymphocytes express either the ligand (AREG) or receptor (EGFR) ex vivo, in sepsis, and expression of CD4^+^ lymphocyte EGFR was associated with several features of immunosuppression. Together, our data suggest that EGFR-signaling represents a novel candidate of T cell dysregulation in sepsis and warrants further investigation.

## Background

We recently discovered that the Epidermal Growth Factor Receptor (EGFR) ligand, amphiregulin (AREG), could identify a cohort of infants with sepsis, even when C-reactive protein (CRP), a prototypic inflammatory marker, was low or normal, identifying it as a potentially adjunctive biomarker for early infection (1). However, whether this immune trait is conserved in adults with sepsis, and the role of AREG-EGFR signalling in sepsis pathophysiology processes including immune suppression, inflammation and tolerance, remain unclear.

AREG and EGFR are critical for epithelial cell proliferation and differentiation, and in prior studies, EGFR-ligands have been implicated in maintaining epithelial barrier defense against bacterial translocation, as well as tissue healing after acute inflammation (2–4). However, there is now a greater appreciation that immune cells can themselves upregulate surface EGFR in situations of inflammation (5). Blockade of this axis and/or genetic deletion of EGFR on immune cells only, can influence outcomes in animal models of cancer, nephritis and high-fat diet (6–8). To this end, we hypothesize that EGFR signalling contributes to immune dysregulation in adult sepsis and sepsis-induced immunosuppression. Here, we examine serum AREG levels and the frequencies of EGFR-expressing myeloid and lymphoid cells in patients with sepsis on the intensive care, comparing sepsis survivors and non-survivors (at 28 days).

## Methods

### Study design and participants

We conducted a prospective observational cohort study of patients aged ≥18 years presenting to the Intensive Care Unit (ICU) at University College London Hospitals (UCLH) between 1^st^ August 2021 and 26^th^ January 2023 who had blood cultures taken for suspicion of bacterial infection. Exclusion criteria for this study included patients with: i) underlying hemato-oncologic diagnoses, ii) severe anemia, ii) a contra-indication to blood transfusion, iii) pregnancy and iv) those not expected to survive beyond 24 hours of admission. Patient demographics, clinical data (physiology, diagnoses), laboratory data, and clinical outcomes were recorded from electronic healthcare records. Patients were followed up to hospital discharge/death. This was a sub-study of a larger study investigating biomarkers in sepsis (REC reference 20/LO/1024). As a reference, venous blood samples were taken from healthy volunteers (HV) between August 2021 and January 2023. Approval was obtained from the University College London (UCL) REC; reference 19181/001.

### Sample processing

Whole blood was collected in CPT™ (8ml), K2 EDTA (4ml), and SST Advance™ vacutainers (4mls, all Becton Dickinson (BD), Wokingham, UK) and processed within 30 minutes. The CPT vacutainers were centrifuged at 1500rcf for 15minutes, the PBMC layer extracted, washed twice in PBS, resuspended in freezing media (fetal bovine serum (FBS) (Gibco, Thermo Fisher (TF), Cambridge, UK) with 10% DMSO (Dimethylsulfoxide, Sigma-Aldrich (SA), Gillingham, UK), and cooled to -80 °C using a Mr Frosty™ isopropyl alcohol gradient (TF). After 24–48 hours, the PBMCs were transferred for long term storage in liquid nitrogen. Frozen PBMCs were rapidly defrosted in batches using media, washed twice in media and rested for 1 hour prior to stimulation. Serum was stored at -80 °C.

### *Ex vivo* PBMC stimulation

Lymphocytes were stimulated with CD3/28 beads (Miltenyi Biotec (MB), Woking, UK) at a concentration of 4:1 (beads: PBMCs) for 72 hours at 37°C, 5% CO_2_ in 11 healthy volunteers and 22 ICU patients.

### Flow cytometry

Cells were acquired an ID7000 spectral cell analyzer (Sony Biotechnology Inc, Weybridge, UK) running ID7000 software (version 1.2, Sony). Alignment check beads were used prior to running each experiment. Spectral references for each fluorochrome were added using either single stain labelled compensation beads (TF) or heat-killed cells (60°C for 10minutes, viability stains) with appropriate negative controls. FMO (fluorescence minus one) samples were used to identify cell populations. The stopping gate was set at 10,000 events for either classical^+^ monocytes or CD4^+^ lymphocytes.

Cells were identified initially by forward and side scatter, singlets (side scatter area by height) and viable. Monocytes were then identified as being HLA-DR^+^, CD11b^-^, CD14^++^/CD16^-^. CD4^+^ lymphocytes were identified as being CD3^+^CD19^-^ and CD4^+^CD8^-^. (**Supplementary Figure 1**).

Fluorochrome panels were designed to investigate cellular immunophenotypes. (**Supplementary Table 1**) Dilutions were determined using dose titrations based on manufacturers recommendations.

### Cytokine measurements

CRP was measured by the hospital laboratory. Serum AREG, IL-1β, IL-6, IL-10, TNF-α, PD-1, and PD-L1 were measured using Duoset ELISA kits (R&D Systems, Abingdon, UK) as per manufacturer instructions. Samples were diluted 1:2 in reagent dilutant. Optical densities were acquired on a SPECTROstar Nano microplate reader (BMG Labtech, Aylesbury, UK).

### Statistical analysis

Statistical analysis was carried out using GraphPAD prism software (Version 10, GraphPad, San Diego, CA). Categorical data was compared using Chi-squared test. For continuous data, a two-tailed Mann Whitney U was used, when two groups were being compared. Where more than two groups were being compared, Kruskal-Wallis test with Dunns uncorrected test for multiple comparisons for three groups, and multivariate analysis was performed and volcano plots generated using a corrected p-value (-log10) with a False Discovery Rate (FDR) of 5% calculated using the two-stage step-up method of Benjamini, Krieger and Yekutieli. The sensitivity and specificity of AREG in predicting mortality was assessed using an area under the receiver operator curve (AUROC). The association of AREG with mortality was assessed by log-rank test and presented as a Kaplan Meier curve.

## Results

### Clinical cohort

We evaluated serum AREG levels in 42 critically ill patients who were admitted to the ICU with sepsis (all patients bacterial infections; 2 with co-existing malaria and one with COVID-19). The median time from ICU admission to blood sampling was 2.5 (1–5) days. A total of 9/42 patients (21%) died in hospital. The median patient age was 57 (range 43-72) and 64% were male (**Table 1**). At ICU admission, Sequential Organ Failure Assessment (SOFA) scores were significantly higher among patients who subsequently died in hospital, compared to those who survived (p=0.004).

**Table 1:** Clinical and biochemical characteristics of ICU population included in serum analysis.

	Total (n=42)	Survivors (n=33)	Non-survivors (n=9)	p-value (survivors *vs* non- survivors)
Age (years)	57 (43–72)	56 (42–72)	57 (53-72)	0.366
Sex (Male)	27/42 (64%)	21/33 (64%)	6/9 (67%)	0.999
Lactate (mmol/l)	1.1 (0.8-2.0)	0.9 (0.7-1.4)	2.2 (1.2-3.6)	0.004*
Creatinine (umol/l)	81 (57-116)	78 (56-108)	98 (56-128)	0.661
Bilirubin (umol/l)	13 (8-30)	13 (5-28)	23 (10-77)	0.081
Platelet count (10^-9^.L)	171 (106-264)	207 (107-275)	112 (73-172)	0.0653
CRP (mg/l)	153 (67-245)	199 (68-248)	79 (22-149)	0.082
White cell count (10^-9^.L)	10.8 (7.5-17.3)	10.6 (7.2-17.3)	11.0 (7.8-18.8)	0.637
Monocyte (10^-9^.L)	0.69 (0.34-1.31)	0.69 (0.33-1.15)	0.62 (0.34-1.11)	0.697
Lymphocyte (10^-9^.L)	1.1 (0.7-1.8)	0.95 (0.66-1.81)	1.39 (0.73-2.57)	0.458
Mechanical ventilation	24/42 (57%)	16/33 (48%)	8/9 (89%)	0.055
Cardiovascular support	16/42 (38%)	10/33 (30%)	6/9 (67%)	0.063
Renal replacement therapy	4/42 (10%)	3/33 (9%)	1/9 (11%)	0.999
SOFA score	6 (4-9)	5 (3-8)	9 (6.5-17)	0.004*
Source of infection				
Blood	2 (5%	2 (6%)	0	0.890
Chest	19 (45%)	14 (42%)	5 (56%)	
CNS	1 (2%)	1 (3%)	0	
ENT	1 (2%)	1 (3%)	0	
Gastrointestinal	16 (38%)	12 (36%)	4 (44%)	
Soft tissue	1 (2%)	1 (3%)	0	
Urological	2 (5%)	2 (6%)	0	

Data expressed as median (interquartile range) or number (percentage) and analyzed using Mann Whitney U or Chi-squared test for continuous of categorical data respectively. * Indicates significant difference (p<0.05). CRP, C-reactive protein; SOFA, Sequential organ failure assessment.

In addition, peripheral blood mononuclear cells (PBMC) were available from a cohort of 35 patients with sepsis, 13 of whom were non-survivors (37%) (**Supplementary Table 2**). This allowed us to apply high-throughput multi-parameter flow cytometry assays, inclusive of EGFR and AREG antibodies, to assess these cellular traits (either as a percentage frequency/median fluorescence intensity (MFI)) between groups. The median patient age was 62 (range 51-71) and 70% were male. ICU admission SOFA sore was significantly higher among non-survivors compared to survivors (p=0.01).

In order to examine baseline cellular expression levels of AREG and EGFR, we included samples from 20 healthy volunteers (HV, 33 (23–40) years, 45% male) (**Supplementary Table 3**).

### Elevated serum AREG levels are associated with mortality in sepsis

**Figure 1:**
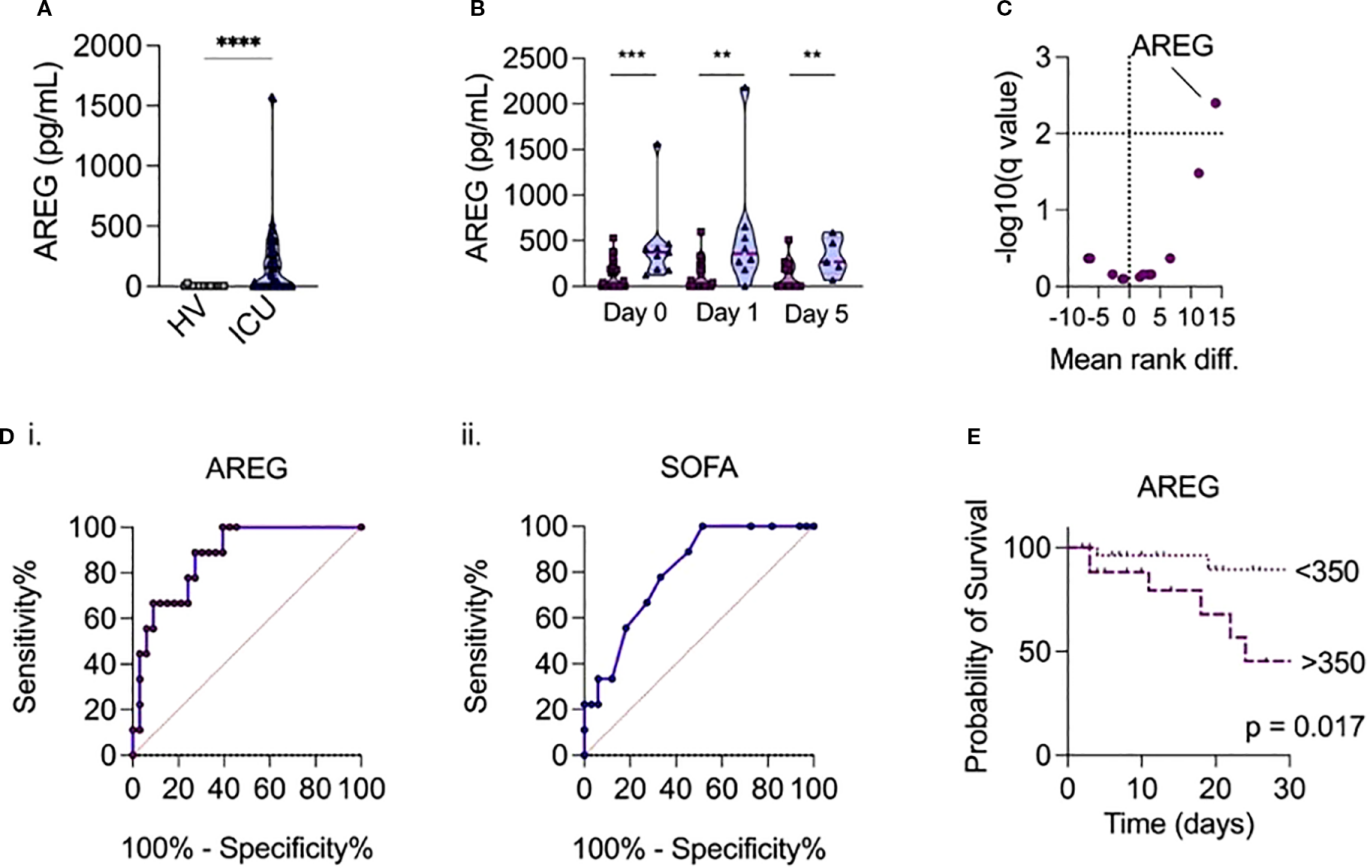
**(a)** Serum AREG levels in patients admitted with sepsis to ICU versus healthy volunteers. **(b)** Temporal measurements of serum AREG in patients with sepsis, according to day of ICU admission, comparing survivors (dark purple) and non-survivors (light purple). P-values were assessed using a univariate two-tailed Mann-Whitney U test (**p < 0.01, ***p < 0.001 add ****p<0.0001). **(c)** Comparison of 15 routinely collected biochemical/hematological variables, selected cytokines and AREG at ICU admission, between sepsis survivors and non-survivors, volcano plot of multivariate analysis generated using multivariate generated using a corrected p-value (-log10) with a False Discovery Rate (FDR) of 5% calculated using the two-stage step-up method of Benjamini, Krieger and Yekutieli. **(di-ii)** Receiver operator curves show relationship between serum AREG and SOFA score and mortality in hospital. **(e)** Patients with serum levels of AREG > 350pg/mL on ICU admission have increased mortality risk compared to patients with serum levels of AREG < 350pg/mL (p=0.017 on log-rank test).

We first examined whether serum AREG levels might be elevated in adult sepsis, compared to healthy donors, as we had previous observed in pediatric infections (1). To that end, we observed that serum AREG levels were significantly higher in patients admitted with infection to ICU compared to healthy volunteers (p<0.0001; **Figure 1a**).

We next compared serum AREG levels between sepsis survivors and non-survivors. Strikingly, we observed that ICU non-survivors had significantly higher levels of AREG on admission (p<0.001), day 1 (p=0.003), and day 5 (p=0.031) compared to survivors (**Figure 1b**). Moreover, when combined with 14 other routinely collected clinical analytes ((CRP, creatinine, lactate, platelet count, lymphocyte count, monocyte count, neutrophil count, leukocyte count) and a panel of *a priori* selected cytokines associated with sepsis (IL-1β, TNF-α, IL-6, IL-10, soluble PD-1, soluble PD-L1)), only serum AREG could differentiate sepsis non-survivors from survivors (mean rank diff 14; -log10 (q value) = 2.4) (**Figure 1c**). Indeed, elevated serum AREG was associated with hospital mortality (AUROC = 0.87, p<0.001), and outperformed the SOFA (Sequential Organ Failure Assessment) score (AUROC = 0.80, p=0.006) (**Figure 1di-ii**). C-reactive protein was not associated with hospital mortality (AUROC = 0.71, p=0.08), implying a potential disconnect between parameters of biochemical inflammation and tissue injury. An AREG cut-off value of 350pg/mL (derived from the Youden’s index) on ICU admission was associated with in-hospital mortality (p<0.05 on log-rank) (**Figure 1e**).

### Lymphocytes are amongst the sources of AREG in sepsis

**Figure 2:**
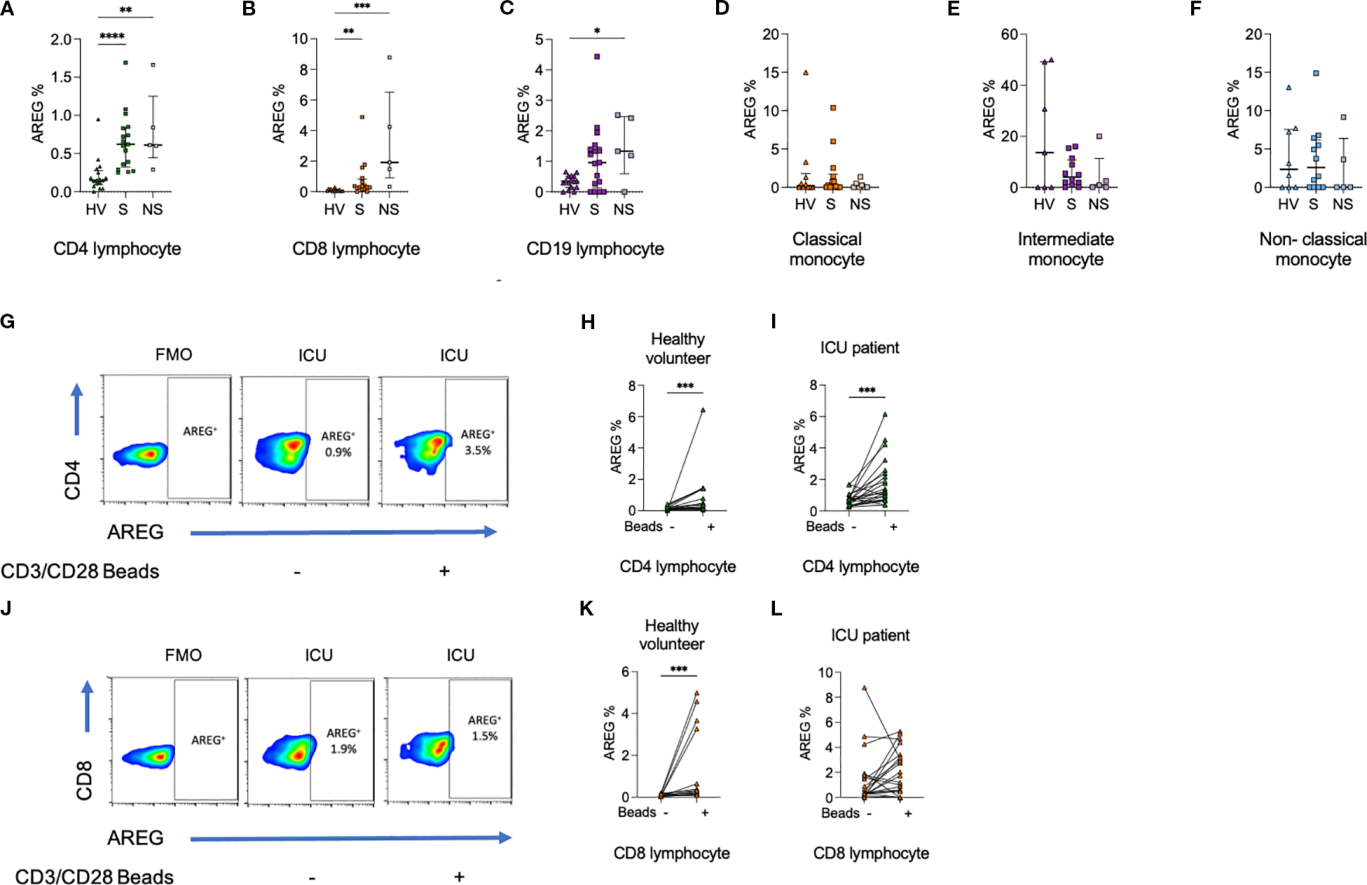
Percentage of **(a)** CD4^+^ lymphocytes, **(b)** CD8^+^ lymphocytes, and **(c)** CD19^+^ lymphocytes expressing AREG are greater among ICU patients compared to HV. In contrast, there was no difference in the percentage of **(d)** classical monocytes, **(e)** intermediate monocytes, or **(f)** non-classical monocytes expressing AREG. **(g–i)**. The percentage of CD4^+^ lymphocytes from HV and ICU patients expressing AREG was increased following exposure to CD3/CD28 beads. **(j–l)**. The percentage of CD8^+^ lymphocytes from HV but not ICU patients expressing AREG was increased following exposure to CD3/CD28 beads. Data compared using Kruskal-Wallis test with uncorrected Dunns test for three groups, or two-tailed Mann Whitney U test for two groups (*p < 0.05, **p < 0.01, ***p < 0.001, ****p<0.0001).

Having identified elevated circulating levels of AREG in sepsis, and higher levels being associated with mortality, we next sought to identify which immune cell types, if any, might be a cellular source of AREG. Thus, we next examined the frequency of AREG^+^ cells in PBMC obtained from healthy volunteers and sepsis patients *ex vivo* and after activation *in vitro* (**Figure 2**). Intriguingly, we observed a modest, yet statistically significant, increase in the frequency of AREG-positive CD4^+^ lymphocytes (p<0.0001) and CD8^+^ lymphocytes (p=0.0002), in ICU patients compared to healthy volunteers. We observed no significant differences between sepsis survivors and non-survivors, nor did we observe any significant differences in the frequency of AREG^+^ cells across any of the groups for myeloid cells (classical monocytes, intermediate monocytes, non-classical monocytes). These data imply that lymphocytes are a prominent source of AREG in the circulation in sepsis.

Similar trends in lymphocyte populations were seen on assessment of MFI, and monocyte populations demonstrated a reduction in AREG MFI in sepsis compared to HV (**Supplementary Figures 2**, **3**). On CD3/CD28 bead stimulation, increases in AREG MFI were seen in CD8^+^ lymphocytes.

### T cells upregulate surface EGFR in sepsis, and is activation- dependent

**Figure 3:**
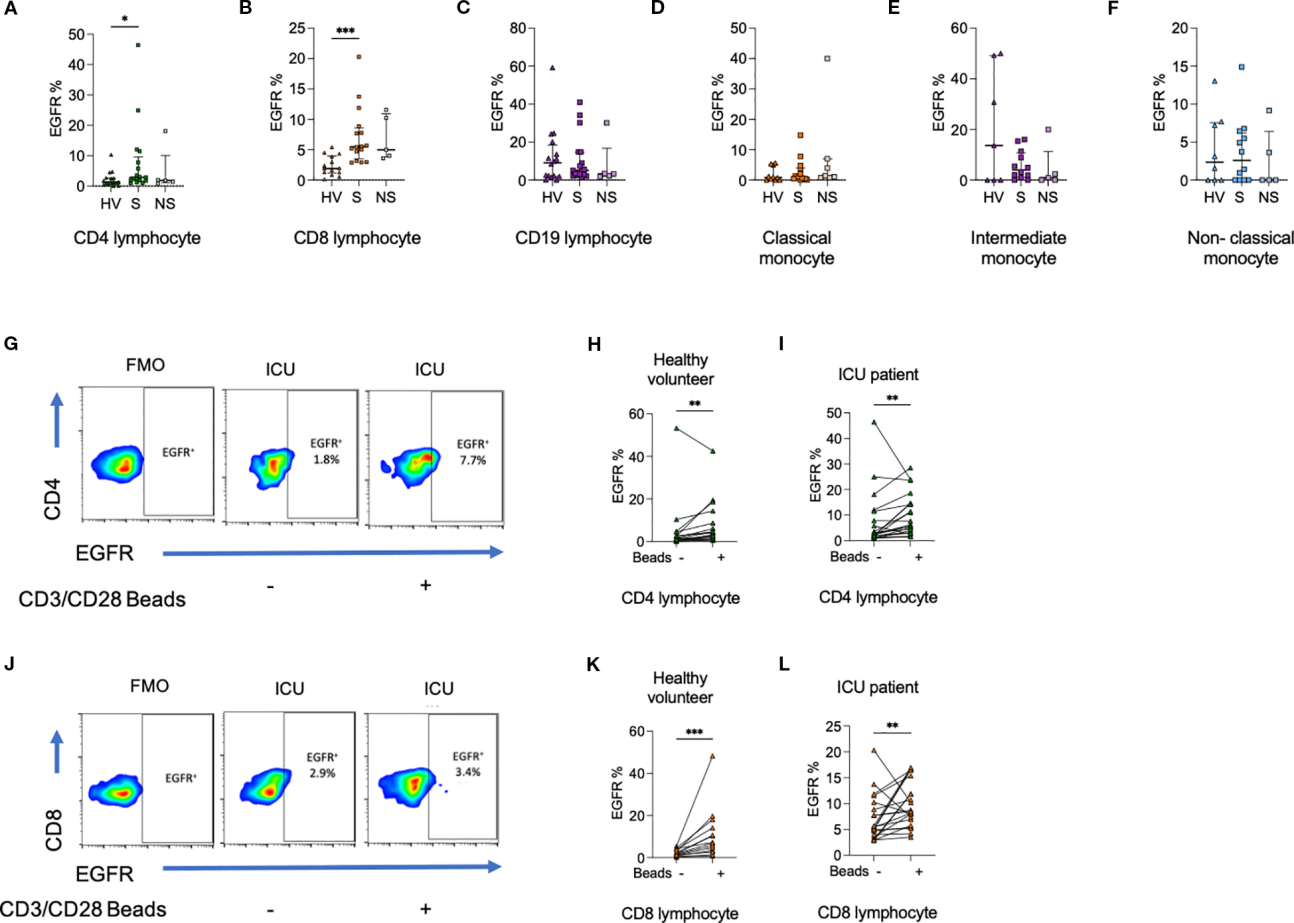
Percentage of **(a)** CD4^+^ lymphocytes, **(b)** CD8^+^ lymphocytes, but not **(c)** CD19^+^ lymphocytes expressing epidermal growth factor receptor (EGFR) are greater among ICU patients compared to HV. There was no difference in the percentage of **(d)** classical monocytes, **(e)** intermediate monocytes, or **(f)** non-classical monocytes expressing EGFR. **(g–i)**. The percentage of CD4^+^ lymphocytes from HV and ICU patients expressing EGFR was increased following exposure to CD3/CD28 beads. **(j–l)**. The percentage of CD8^+^ lymphocytes from HV and ICU patients expressing EGFR was increased following exposure to CD3/CD28 beads. Data compared using Kruskal-Wallis test with uncorrected Dunns test for three groups, or two-tailed Mann Whitney U test for two groups (*p < 0.05, **p < 0.01, ***p < 0.001).

To establish whether circulating immune cells in adult septic patients could directly be influenced by AREG, a prerequisite to our hypothesis that EGFR-axis signaling contributes to sepsis immune dysregulation, we assessed surface levels of EGFR in healthy volunteers and patient PBMCs. The frequency of CD4^+^ lymphocytes (p<0.05), CD8^+^ lymphocytes (p<0.0001) expressing EGFR was higher among ICU patients compared to healthy volunteers. No differences in EGFR expression were seen in CD19^+^ lymphocytes, or monocyte subsets (**Figure 3**).

Given that we observed higher T cell frequencies of EGFR expression in sepsis versus healthy volunteers, we hypothesized that EGFR-expression within lymphocytes may require prior cellular activation. To recapitulate this *in vitro*, we next exposed HV or ICU sepsis patient PBMCs to CD3/CD28 beads and assessed changes in EGFR percentage and MFI from baseline. We observed an increase in the percentage of CD4^+^ (p<0.01) and CD8^+^ (p<0.001) lymphocytes expressing EGFR (**Figure 3**).

On assessment of MFI, there were no differences in EGFR expression between HV and ICU patients (**Supplementary Figures 4**, **5**). However, changes to CD4^+^ and CD8^+^ lymphocyte EGFR MFI on CD3/CD28 stimulation were similar to changes in percentage positive cells.

### Associations between EGFR and immunophenotype of CD4^+^ and CD8^+^ lymphocytes in sepsis

**Figure 4:**
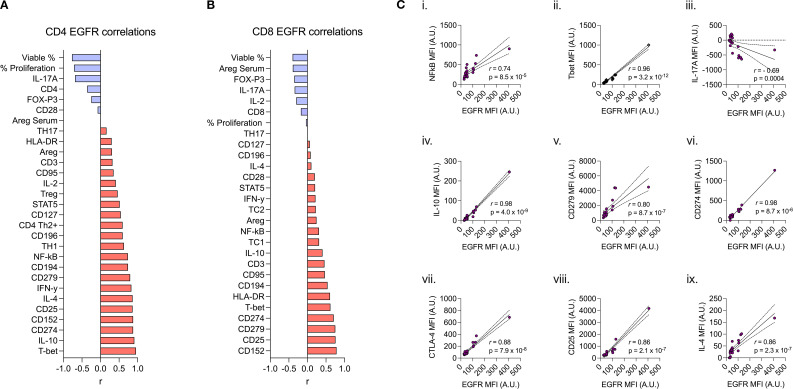
Spearman correlation of **(a)** CD4^+^ and **(b)** CD8^+^ lymphocyte expression of EGFR (MFI) to 27 other immunophenotypic markers. **(ci-ix)** Spearman rank correlation scatter plots comparing CD4+ expression of EGFR (MFI) by specific biomarkers (correlation coefficient (r), p values denoted on graphs) **(c.i)** NFkB, **(c.ii)** Tbet, **(c.iii)** IL-17A, **(c.iv)** IL-10, **(c.v)** CD279, **(c.vi)** CD274, **(c.vii)** CTLA-4, **(c.viii)** CD25, and **(c.ix)** IL-4.

Given the capacity for CD4^+^ and CD8^+^ lymphocytes to upregulate AREG/EGFR expression upon activation, we next assessed correlations between these markers and other prototypic indices of cellular activation, exhaustion or functional capacity. CD4^+^ lymphocyte EGFR expression positively correlated with cellular traits including: CD279 (PD-1) (*r*=0.80; p=8.7x10^-7^), CD25 (IL-2R) (*r*=0.86; p=2.1x10^-7^), Tbet (*r*=0.96; p=3.2x10^-12^), NFκB (r=0.74, p=8.5x10^-5^)and CD152 (CTLA-4) (*r*=0.88, p=7.9x10^-8^) as well as cytokines IL-10 (r=0.91; p=4.0x10^-9^), IL-4 (r=0.86; p=2.3x10^-7^) and IFN-γ (r=0.83; p=1.7x10^-6^). In contrast, CD4^+^ lymphocyte EGFR expression negatively correlated with IL-17A (*r*=-0.69; p=0.0004) (**Figure 4**). AREG expression correlated with CD4^+^ lymphocyte CD95 (r=0.50; p<0.05), and CD279 (r=0.55; p<0.01) (**Supplementary Figure 6a**).

In CD8^+^ lymphocytes, EGFR expression correlated with CD152 (r=0.79; p=2.8x10^-5^), CD279 (r=0.75; p=2.5x10^-4^), CD274 (PD-L1) (r=0.71; p=3.2x10^-6^), Tbet (r=0.62; p=9.2x10^-5^), HLA-DR (r=0.61; p<0.01), and CD194 (CCR4) (r=0.54; p<0.05) (**Figure 4**). There were no correlations between CD8^+^ lymphocyte AREG and other functional proteins (**Supplementary Figure 6b**).

## Discussion

Our study of critically ill adults with sepsis highlights the strong association between serum AREG and in-hospital mortality. Moreover, we demonstrate that a higher frequency of circulating CD4^+^ and CD8^+^ lymphocytes express either the ligand (AREG) or receptor (EGFR) *ex vivo*, in sepsis, suggesting that EGFR-signalling represents a novel candidate of T cell dysregulation in sepsis.

Our data are supported by reports in other infection settings such as COVID-19, where AREG levels in serum were shown to be elevated in critical versus non-critical COVID-19, and in a smaller validation cohort, the authors observed a non-significant trend towards elevation of AREG in non-survivors versus survivors of sepsis (9). Nonetheless, our data add significant value to this prior evidence, given our findings that AREG alone, but not any routine clinical test (e.g., CRP) or pro-inflammatory cytokine (e.g., IL-6) had the ability to differentiate non-survivors, as early as at ICU admission, identifying its potential utility as a marker of poor prognosis in sepsis.

Whilst the EGFR axis has been extensively studied in humans, the majority of data focus on its role in mesenchymal cell biology, with relevance to tissue barrier integrity, wound healing and dysregulation of this axis, leading to epithelial cancers (8). In contrast, relatively little is known about whether, and to what extent, EGFR signalling might influence immune cell phenotype and function under physiological conditions and in during perturbed states, such as sepsis. Here, we identify a modest, yet significant, increase in the *ex vivo* frequencies of EGFR-expressing T cells in the peripheral blood of septic patients, though curiously this was not the case in the other cell types assessed, including B cells, or monocytes/dendritic cells.

AREG an anti-inflammatory phenotype in lymphocytes, mediated via T_reg_ responses (10), which may have a beneficial role in the resolution of inflammation in sepsis. In murine models of influenza infection, administration of recombinant AREG improved lung function through bronchial repair and regeneration, resulting in reduced mortality (3). CD4^+^ lymphocyte EGFR levels related to IL-4, the latter which mediates T_reg_-mediated immune suppression (11). Others have described similar findings, with decreased OKT-induced CD4^+^ and CD8^+^ lymphocyte proliferation, increased cell death and increased IL-10 in the presence of AREG (12). In Hepatitis B infection, AREG promotes the immunosuppressive activity of intrahepatic T_reg_ cells to impair CD8^+^ lymphocyte immunity against hepatitis B virus infection (13).

An immunosuppressed CD4^+^ lymphocyte phenotype including increased immune checkpoint inhibition (increased PD-1), impaired lymphocyte proliferation/maturation (reduced IL-7 receptor), inhibition (increased CTLA-4), and decreased viability are associated with secondary infections and mortality in sepsis (14, 15). We found that CD4^+^ lymphocyte EGFR expression was associated with elevated expression of IL-10, PD-1, CTLA-4, and decreased IL-17A, and viability. Our data, supported by the literature, raise the possibility that activation of the AREG-EFGR axis may contribute to the immunosuppressed CD4^+^ lymphocyte phenotype in sepsis and risk of infectious complications.

In contrast to lymphocytes, activation of the AREG-EGFR axis may promote inflammation in monocytes and macrophages via TLR-4 activity. Although the increase in AREG and EGFR expression in sepsis was evident in CD4^+^ and CD8^+^ lymphocytes, AREG and EGFR expression was greatest in intermediate monocytes; consistent with the reported literature (1, 12). EGFR is required for monocyte/macrophage TLR4-mediated activation of NFκB in response to lipopolysaccharide (LPS) (16). Inhibition of the AREG-EGFR axis with erlotinib is associated with improved survival in mouse models of endotoxemia (16, 17). In mice, erlotinib reduces both cytokine expression and toxicity in response to LPS, suggesting that EGFR inhibitors may be of use in treating septic shock. Additionally, inhibition of surface TLR4 expression by Erlotinib alleviates macrophage parthanatos (PARP-1 dependent cell death) in response to LPS (17). We did not find any differences in EGFR expression in monocytes in health and sepsis.

Together, experimental data suggest that inhibition of the AREG-EGFR axis could be beneficial in sepsis, via reduced monocyte/macrophage – mediated inflammation and enhanced CD4^+^ and CD8^+^ effector cell functions. However, observational clinical data suggest that use of small molecule tyrosine kinase inhibitors of EGFR (e.g., erlotinib) for lung cancer is associated with increased risk of developing infectious complications (18), although guidelines do not yet advocate for the use of antimicrobial prophylaxis in patients receiving these therapies (19). Additionally, inhibition of the AREG-EFGR axis may impair resolution of inflammation. The data we present are correlative and do not imply causality. However, the striking association between elevated AREG levels and mortality in sepsis warrants further mechanistic studies evaluating the therapeutic benefit of modulating the AREG-EGFR axis. Particular emphasis should be placed on the timing of intervention in relation to illness onset, risk of secondary infections, and resolution in inflammation.

We have not performed sequential measurements to assess the trajectory of immune cell function over time. The variable timing from sepsis onset to ICU presentation reflects real-world data, which may influence findings. However, we provide temporal data on the association of serum AREG levels in survivors and non-survivors, which strengthen our findings. All *in vitro* experiments were performed using a single concentration of CD3/CD28 beads, and the response to an *in vitro* stimulus may not represent *in vitro* changes in patients with infections. We have not investigated the AREG-EGFR axis in other cell types impaired in sepsis, including neutrophils (20, 21). Neutrophils are known to be inducers of AREG in non-immune cells, in part through release of neutrophil elastase, and may induce therefore induce AREG in immune cells given their role in activation of the adaptive immune system (22). We have not presented data on lymphocyte subsets as cell counts from patients were limited.

Several studies have assessed the transcriptomic profile of immune cells in sepsis, although transcriptional changes may not be reflected in cell surface proteins/receptor expression, and bulk transcriptomics do not directly assess the phenotype of specific cell subsets. Flow cytometry has allowed the assessment of single cell protein expression.

Another strength of our study is the assessment of dynamic immune function (responses to stimulus or treatment), in addition to basal immune phenotype. Although the expression of cell surface receptors is often used as a surrogate of cell activation/function, the ability to respond to further stimuli remains an important facet in the context of secondary infections. We therefore evaluated *ex vivo* lymphocyte response to CD3/CD28 beads.

In summary, we provide novel data on the striking association between elevated AREG levels and mortality in sepsis. The AREG-EGFR axis orchestrates both inflammation and resolution. Inhibition of the AREG-EGFR axis may have the dual benefit of simultaneously ameliorating monocyte-induced systemic inflammation whilst promoting CD4^+^ lymphocyte activity. The AREG-EGFR axis as a theragnostic target warrants further evaluation.

## Data Availability

The raw data supporting the conclusions of this article will be made available by the authors, upon reasonable request.

## Group members of University College London Hospitals Critical Care Research Team

Naveed Saleem, Antonio Cesar, Alessia V. Waller, Francis Ryckaert, Deborah Smyth, Georgia Bercades, Ingrid Hass, Laura Gallagher, and Gladys Martir.
