# Supplementary material for: The amphiregulin- epidermal growth factor receptor axis as a therapeutic target in sepsis
Source: Front Immunol. 2025 Sep 23;16:1638244. doi: 10.3389/fimmu.2025.1638244 (PMC12500437; doi:10.3389/fimmu.2025.1638244)
Supplement: Supplementary file 1 [file DataSheet1.docx]

Supplementary Tables

| **Function** | **Marker type** | **Cell Marker** | **Fluorochrome** | **Cat no** | **Species** | **Isotope** | **Dilution** |
| --- | --- | --- | --- | --- | --- | --- | --- |
| **All panels** | | | | | | | |
| Epidermal growth factor pathway | Intracellular protein | Amphiregulin | PE | TF 12-5370-42 | Mouse | IgG1, κ | 1:100 |
|  | Cell surface | EGFR | BUV661 | BD 750827 | Mouse | IgG1, κ | 1:250 |
| Viability | Viability | Live-Dead | Zombie NIR | BL 423106 | - | - | 1:1000 |
| **Myeloid cells panel** | | | | | | | |
| Cell identification | Cell surface | CD11b | SBUV445 | BR MCA711 | Rat | IgG2b | 1:250 |
|  | Cell surface | CD14 | SBB580 | BR MCA1568 | Mouse | IgG2a | 1:250 |
|  | Cell surface | CD16 | SB702 | TF 67-0168-42 | Mouse | IgG1, κ | 1:250 |
|  | Cell surface | HLA-DR | BUV805 | BD 748338 | Mouse | IgG2a, κ | 1:250 |
| **Lymphocyte panel** | | | | | | | |
| Cell identification & activation | Cell surface | CD3 | SBUV445 | BR MCA463 | Mouse | IgG1 | 1:250 |
|  | Cell surface | CD4 | BUV805 | TF 368-0047-42 | Mouse | IgG1, κ | 1:250 |
|  | Cell surface | CD8 | APC-Fire 750 | BL 301066 | Mouse | IgG1, κ | 1:250 |
|  | Cell surface | CD19 | BUV395 | TF 363-0198-42 | Mouse | IgG1, κ | 1:250 |
| Proliferation | Proliferation | CellTrace | FarRed | TF C34564 | - | - | 1:1000 |
|  | Cell surface | CD127 | PerCP-eFluor 710 | TF 46-1278-42 | Mouse | IgG1, κ | 1:250 |
| Differentiation | Cell surface | IL-2R (CD25) | SBV570 | BR MCA2127 | Mouse | IgG1 | 1:250 |
|  | Cell surface | CCR4 (CD194) | BUV563 | BD 752566 | Mouse | IgG1, κ | 1:250 |
|  | Cell surface | CCR6 (CD196) | BV786 | BD 563704 | Mouse | IgG1, κ | 1:250 |
|  | Intracellular cytokine | IL-2 | BV650 | BL 500334 | Rat | IgG2a, κ | 1:100 |
|  | Intracellular cytokine | IL-4 | PE-Cy7 | BD 560672 | Mouse | IgG1, κ | 1:100 |
|  | Intracellular cytokine | IL-17A | APC-R700 | BD 565163 | Mouse | IgG1, κ | 1:100 |
|  | Intracellular protein | STAT5 | RB780 | BD 568759 | Mouse | IgG1, κ | 1:100 |
|  | Transcription factor | Fox-p3 | PE-Cy5 | TF 15-4776-42 | Rat | IgG2a, κ | 1:100 |
|  | Transcription factor | T-bet | BV605 | BL 644817 | Mouse | IgG1, κ | 1:100 |
| Activation | Cell surface | CD28 | BUV496 | BD 741168 | Mouse | IgG1, κ | 1:250 |
|  | Cell surface | HLA-DR | BV711 | BD 563696 | Mouse | IgG2a, κ | 1:250 |
|  | Intracellular protein | NF-κB | PE-CF594 | BD 565447 | Mouse | IgG2b, κ | 1:100 |
| Suppression | Cell surface | CTLA-4 (CD152) | AF532 | BT NBP2-50286 | Mouse | IgG1, κ | 1:250 |
|  | Cell surface | PD-L1 (CD274) | FITC | BL 393606 | Mouse | IgG1, κ | 1:250 |
| Cell death | Cell surface | Fas (CD95) | BUV615 | BD 752346 | Mouse | IgG1, κ | 1:250 |
|  | Cell surface | PD-1 (CD279) | BV480 | BD 566112 | Mouse | IgG1, κ | 1:250 |
| Cytokine release | Intracellular cytokine | IFN-γ | BV750 | BD 566357 | Mouse | IgG1, κ | 1:100 |
|  | Intracellular cytokine | IL-10 | BUV737 | TF 367-7108-42 | Rat | IgG1, κ | 1:100 |

**Supplementary Table 1. Spectral flow cytometry fluorochromes**

Abbreviations: APC: Allophycocyanin, AF: Alexa Fluor, BD: Beckton Dickinson, BL: Biolegend, BR: Biorad, BT: Biotechne, BUV: Brilliant ultraviolet, BV: Brilliant violet, CD: cluster of differentiation, CF: Cyanine-based fluorescent dye, CTLA-4: Cytotoxic T-lymphocyte associated protein-4, Cy: Cyanine, EGFR: Epidermal growth factor receptor, FITC: Fluorescein isothiocyanate, Fox-P3: Forkhead box P3, IFN: Interferon, IL: Interleukin, L/D: Live/Dead, MB: Miltenyi Biotec, NF-κB: Nuclear Factor Kappa B, NIR: Near-infrared, PD-1: Programmed death receptor 1 PD-L1: Programmed death-ligand 1, PE: Phycoerythrin, PerCP: Peridinin-chlorophyll-protein, RB: RealBlue, SBV: StarBright violet, SBUV: StarBright ultraviolet, STAT5: Signal transducer and activator of transcription 5, T-bet: T-box transcription factor TBX21, TF: Thermo Fischer, UV: Ultraviolet.

|  | | Total  (n=35) | Survivors  (n=22) | Non-survivors  (n=13) | p-value  (survivors vs non- survivors) |
| --- | --- | --- | --- | --- | --- |
| Age (years) | | 62 (51-71) | 64.5 (56.25 - 72) | 59 (49-69) | 0.7157 |
| Sex (Male) | | 25 (70%) | 15 (68%) | 10 (77%) | 0.7094 |
| Lactate (mmol/l) | | 0.8 (0.7-1.3) | 0.8 (0.7 - 1.0) | 1.1 (0.8 -1.5) | 0.0981 |
| Creatinine (umol/l) | | 63 (45-88) | 64 (50 - 80) | 69 (40 - 100) | 0.9618 |
| Bilirubin (umol/l) | | 11.5 (6.8-17.5) | 10 (6 -14) | 16 (12 - 28) | 0.0240* |
| Platelet count (10^-9^.L) | | 187 (64-240) | 215 (136 - 265) | 149 (47 - 190) | 0.0494* |
| CRP (mg/dl) | | 140 (78-252) | 141 (84 - 231) | 148 (76- 260) | 0.8262 |
| White cell count (10^-9^.L) | | 9.3 (4.3-12.6) | 9.7 (7.3- 12.1) | 6.6 (3.4-17) | 0.5015 |
| Monocyte (10^-9^.L) | | 0.68 (0.28-01.8) | 0.68 (0.27 - 1.06) | 0.58 (0.18 -0.98) | 0.5581 |
| Lymphocyte (10^-9^.L) | | 0.88 (0.49-1.43) | 1.5 (0.79 - 1.5) | 0.56 (0.21 – 0.87) | 0.0122* |
| SOFA score | | 6 (5-8) | 5 (3 - 8) | 8 (6 - 10) | 0.0095* |
| Source of infection | | | | | |
|  | Blood | 1 (2%) | 0 | 1 (8%) | 0.384 |
|  | Chest | 19 (54%) | 11 (50%) | 8 (62%) |  |
|  | CNS | 1 (2%) | 0 | 1 (8%) |  |
|  | ENT | 1 (2%) | 1 (5%) | 0 |  |
|  | Gastrointestinal | 9 (26%) | 7 (32%) | 2 (15%) |  |
|  | Soft tissue | 2 (6%) | 1 (5%) | 1 (8%) |  |
|  | Urological | 2 (6%) | 2 (9%) | 0 |  |

**Supplementary Table 2.** Clinical and biochemical characteristics of ICU population included in PBMC analysis

Data expressed as median (interquartile range) or number (percentage) and analysed using Mann Whitney U or Chi-squared test for continuous of categorical data respectively. * indicates significant difference (p<0.05). Abbreviations: CRP: C-reactive protein, SOFA: Sequential organ failure assessment.

|  | Total  (n=20) |
| --- | --- |
| Age (years) | 33 (23-40) |
| Sex (Male) | 9 (45%) |
| Ethnicity | |
| Asian | 10 (50%) |
| Black | 2 (10%) |
| White | 8 (40%) |

**Supplementary Table 3.** Clinical characteristics of healthy volunteers

Data expressed as median (interquartile range) or number (percentage).

# Supplementary Figures


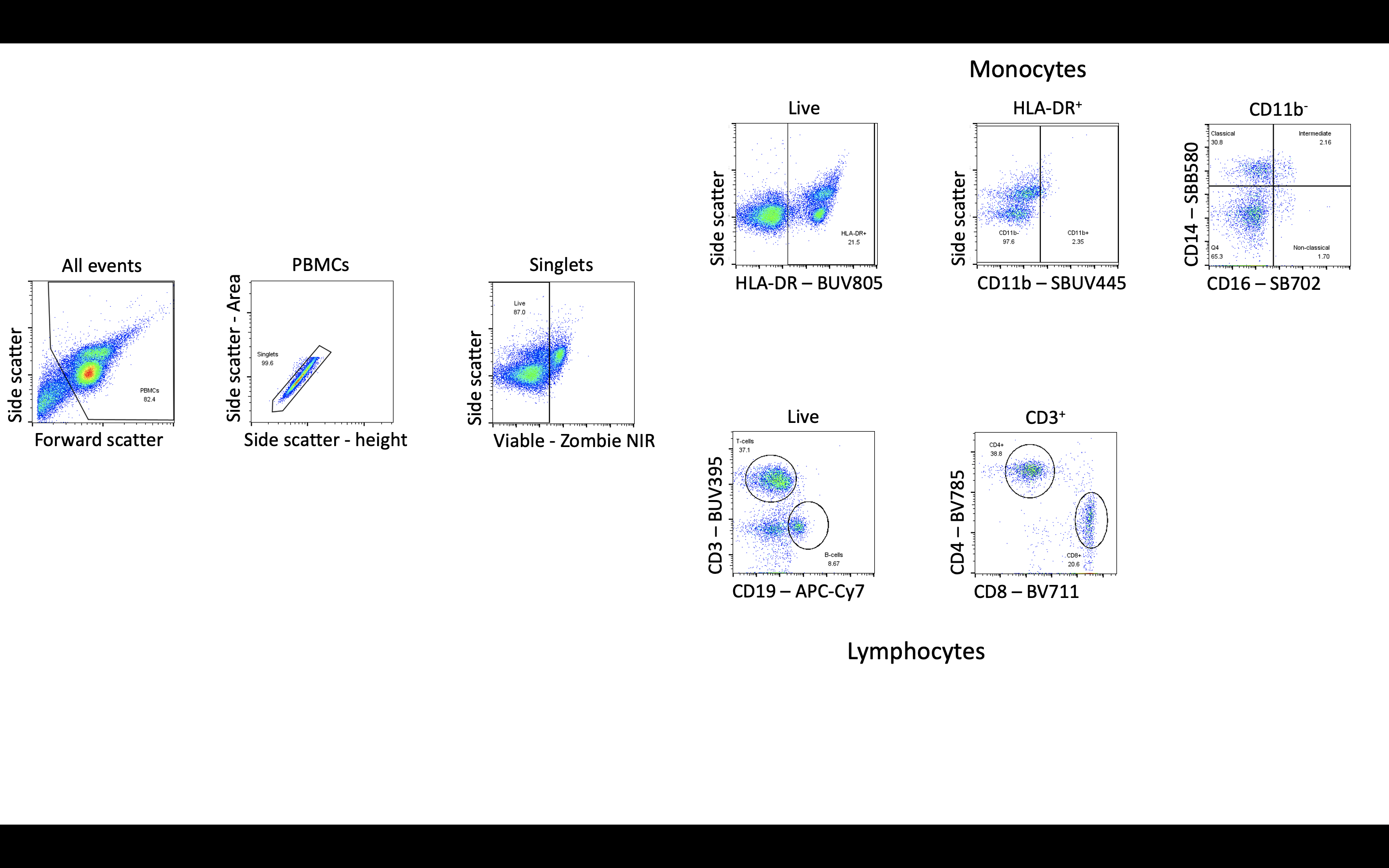


**Supplemental Figure 1: Gating strategy for classical monocytes and CD4^+^ and CD8^+^ lymphocytes**

PBMCs were gated initially on forward and side scatter, singlets then identified using side scatter area by height, and then viability using Zombie near infrared (NIR). Monocytes (top) were then identified as HLA-DR^+^ (using Brilliant UV (BUV) 805), CD11b^-^ (using StarBright Ultraviolet (SBUV) 402), and finally CD14^++^/CD16^-^ (using StarBright Blue (SBB) 580 and SuperBright (SB) 702). Lymphocytes (bottom) were differentiated using CD3^+^ or CD19^+^ (using BUV395 and Allophycocyanin-Cyanine7 (APC-Cy7), then CD4^+^ or CD8^+^ (Brilliant violet (BV) 785 and BV711).

**Supplemental Figure 2**: Expression of AREG in lymphocyte and monocyte populations in healthy volunteers and ICU patients. (MFI: median fluorescence units), A.U. (arbitrary units). Data compared using Kruskal-Wallis test with Dunns uncorrected test for multiple comparisons. *(*p < 0.05, **p < 0.01, ***p < 0.001)*

**Supplemental Figure 3**: Expression of AREG in lymphocyte populations in healthy volunteers and ICU patients before and after stimulation with CD3/CD28 beads. (MFI: median fluorescence units), A.U. (arbitrary units). Data compared using two tailed Mann Whitney U test. *(*p < 0.05, **p < 0.01, ***p < 0.001)*

**Supplemental Figure 4**: Expression of EGFR in lymphocyte and monocyte populations in healthy volunteers and ICU patients. (MFI: median fluorescence units), A.U. (arbitrary units). Data compared using Kruskal-Wallis test with Dunns uncorrected test for multiple comparisons. *(*p < 0.05, **p < 0.01, ***p < 0.001)*

**Supplemental Figure 5**: Expression of EGFR in lymphocyte populations in healthy volunteers and ICU patients before and after stimulation with CD3/CD28 beads. (MFI: median fluorescence units), A.U. (arbitrary units). Data compared using two tailed Mann Whitney U test. *(*p < 0.05, **p < 0.01, ***p < 0.001)*


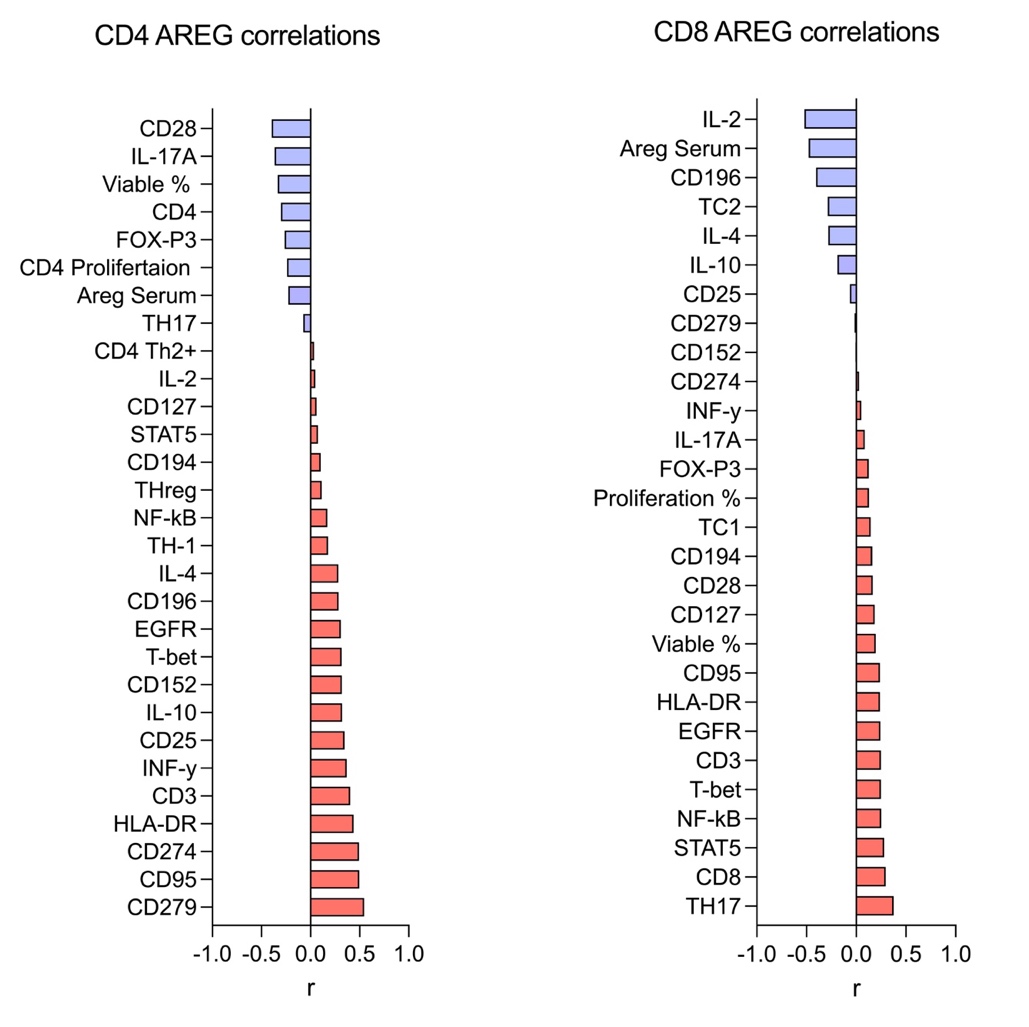


**Supplemental Figure 6**: Spearman correlation of (a) CD4^+^ and (b) CD8^+^ lymphocyte expression of AREG (MFI) to 27 other phenotypic markers
